# Association between triglyceride–glucose and fibrosis-4 indices and nephrolithiasis: a cross-sectional case–control study

**DOI:** 10.1590/1806-9282.20260298

**Published:** 2026-10-12

**Authors:** Sezin Doğan Çakır, Mustafa Erkoç, Mine Adaş

**Affiliations:** 1 University of Health Sciences, Prof. Dr. Cemil Taşcıoğlu City Hospital, Department of Endocrinology – Istanbul, Turkey.; 2 University of Health Sciences, Prof. Dr. Cemil Tascıoğlu City Hospital, Department of Urology – Istanbul, Turkey.

**Keywords:** Nephrolithiasis, Insulin resistance, Triglycerides, Blood glucose, Biomarkers

## Abstract

**OBJECTIVE:**

Fibrosis-4 (a liver fibrosis index) and triglyceride–glucose (an insulin resistance surrogate) indices may be markers associated with kidney stone risk. The aim of this study was to evaluate the association between triglyceride–glucose and fibrosis-4 indices and the presence of nephrolithiasis in adults.

**METHODS:**

This retrospective cross-sectional case–control study included 918 adults (785 with nephrolithiasis, 133 controls) evaluated between January 2016 and December 2023. Controls were consecutive patients from the same urology outpatient population who underwent abdominal imaging for non-stone-related urological indications, with no radiological evidence of nephrolithiasis. Triglyceride–glucose and fibrosis-4 indices were calculated using standard formulas. The triglyceride–glucose index was standardized (per 1 SD=0.23 units) prior to multivariable logistic regression including age, sex, and fibrosis-4.

**RESULTS:**

Triglyceride–glucose was significantly higher in stone formers (4.87±0.21 vs. 4.64±0.20; p<0.001). In multivariable analysis, triglyceride–glucose independently predicted nephrolithiasis (adjusted OR per 1 SD=3.52; 95%CI 2.48–5.00; p<0.001); male sex was also independently associated. Fibrosis-4 was not independently associated (p=0.809). Triglyceride–glucose area under the curve was 0.779 (95%CI 0.729–0.827), consistent across both sexes (interaction p=0.135).

**CONCLUSION:**

Each 1 SD increase in triglyceride–glucose was associated with a 3.5-fold increase in odds of nephrolithiasis, independent of age, sex, and fibrosis-4. Fibrosis-4 showed no independent utility. Prospective studies are needed to confirm these findings.

## INTRODUCTION

Nephrolithiasis affects 10–15% of adults worldwide and poses a growing clinical and economic burden^1^. Increasing evidence supports the concept of kidney stone disease as a systemic metabolic condition rather than an isolated urological disorder^2,3^. Metabolic syndrome and its components, particularly insulin resistance and diabetes, have been consistently associated with nephrolithiasis in population-based studies and meta-analyses, reinforcing a systemic metabolic basis for stone formation^4,5^.

Metabolic dysfunction-associated steatotic liver disease (MASLD) has similarly emerged as a major global health concern, currently affecting more than one-third of adults, with prevalence projected to exceed 55% by 2040^6^. Meta-analyses have confirmed an association between MASLD and nephrolithiasis, suggesting a shared metabolic and inflammatory pathophysiology linking hepatic and renal manifestations of insulin resistance^7,8^.

The triglyceride–glucose (TyG) index, derived from fasting triglyceride and glucose levels, is a validated surrogate marker of insulin resistance and has been associated with nephrolithiasis^9,10^. The fibrosis-4 (FIB-4) index, calculated using age, aminotransferases, and platelet count, is a first-line noninvasive tool for assessing liver fibrosis in MASLD and reflects cumulative metabolic burden^11^. Data evaluating FIB-4 in stone formers remain limited. This study aimed to assess the association between TyG and FIB-4 indices and nephrolithiasis in adults.

## METHODS

### Study design and population

This retrospective cross-sectional case–control study was conducted at Prof. Dr. Cemil Taşcıoğlu City Hospital. Adults (≥18 years) who underwent abdominal imaging in the urology department between January 2016 and December 2023 were screened. All consecutive eligible patients meeting the inclusion criteria were included. Patients with radiologically confirmed nephrolithiasis constituted the stone group. Controls were selected from the same source population and consisted of consecutive patients who underwent abdominal ultrasonography and/or computed tomography for non-stone-related urological indications, including microscopic hematuria, non-specific flank pain, lower urinary tract symptoms, simple renal cysts, varicocele, or hydrocele, with no imaging evidence of nephrolithiasis.

Participants with complete metabolic laboratory and imaging data were included. Exclusion criteria applied to both groups were chronic viral or autoimmune liver disease, diabetes mellitus, pregnancy, malignancy, chronic kidney disease, active infection (including urinary tract infection, pyelonephritis, and prostatitis), clinically significant urinary obstruction, and incomplete records. Given the retrospective nature of the study and the use of de-identified patient data, the ethics committee granted a waiver of individual informed consent, in accordance with institutional policy and the Declaration of Helsinki. The study was approved by the local ethics committee (Approval date: 20.11.2023; Approval number: 273).

### Data collection

Demographic characteristics, comorbidities, and fasting laboratory parameters (triglycerides, glucose, aspartate aminotransferase (AST), alanine aminotransferase (ALT), and platelet count) were obtained from electronic medical records. Abdominal imaging included non-contrast computed tomography and/or ultrasonography. The presence of hepatic steatosis was recorded based on standard radiologic criteria.

### Index calculations

FIB-4=(Age×AST) / [Platelet count×√ALT]^11^. TyG=ln [fasting triglycerides (mg/dL)×fasting glucose (mg/dL)/2]^9^.

### Statistical analysis

Continuous variables were expressed as mean±SD, and categorical variables as frequencies and percentages. Normality was assessed using the Kolmogorov-Smirnov test. Triglycerides and FIB-4 were non-normally distributed and were compared using the Mann-Whitney U test; other variables were compared using independent samples t-tests. Categorical variables were compared using chi-square tests. Multivariable logistic regression was performed using the enter method with age, sex, TyG index, and FIB-4 index entered simultaneously. The TyG index was standardized (z-score: [TyG-mean]/SD) prior to analysis to yield odds ratios per 1 SD. Sex-stratified analyses and a sex×TyG interaction term were tested. Receiver operating characteristic (ROC) curve analysis evaluated discriminative performance of TyG; area under the curve (AUC) was calculated with 95%CIs (bootstrap, 2000 iterations). Statistical significance was p<0.05 (two-tailed). SPSS version 25.0 (IBM Corp., Armonk, NY, USA) was used.

## RESULTS

### Baseline characteristics

A total of 918 individuals were included (785 stone formers, 133 controls). Age did not differ significantly between groups (45.77±11.55 vs. 44.29±9.88 years; p=0.161). Male sex was more prevalent among stone formers (69.6 vs. 37.6%; p<0.001). Comorbidities were comparable (Table 1).

**Table 1:** Baseline demographic and clinical characteristics.

Variable	Nephrolithiasis (n=785)	Controls (n=133)	p-value
Age (years)	45.77±11.55	44.29±9.88	0.161
Male sex, n (%)	546 (69.6%)	50 (37.6%)	<0.001
Hypertension, n (%)	13.1%	13.2%	0.946
Cardiovascular disease, n (%)	1.4%	0.0%	0.553
Inflammatory/rheumatologic disease, n (%)	0.4%	0.0%	0.763

Continuous variables: mean±SD; categorical: n (%).

### Laboratory parameters and metabolic indices

The TyG index was significantly higher in the nephrolithiasis group (4.87±0.21 vs. 4.64±0.20; p<0.001), driven by markedly higher fasting triglyceride levels (189.16±70.11 vs. 127.31±47.02 mg/dL; p<0.001). Fasting glucose did not differ significantly (p=0.101). The FIB-4 index was modestly elevated in stone formers (0.83±0.38 vs. 0.77±0.27; p=0.040); however, both values are well within the low-risk range for advanced hepatic fibrosis (<1.45), and this difference did not survive Bonferroni correction (adjusted threshold ≈0.006 for eight comparisons). Aminotransferases and platelet counts were similar between groups (Table 2).

**Table 2:** Laboratory parameters and metabolic indices by group.

Variable	Nephrolithiasis (n=785)	Controls (n=133)	p-value
Fasting glucose (mg/dL)	96.2±39.4	90.6±7.7	0.101
Triglycerides (mg/dL)	189.16±70.11	127.31±47.02	<0.001
TyG index	4.87±0.21	4.64±0.20	<0.001
AST (U/L)	19.8±6.3	19.2±4.8	0.311
ALT (U/L)	20.7±9.3	19.6±6.5	0.118
Platelet count (×10^9^/L)	261.11±54.21	258.77±45.08	0.593
FIB-4 index	0.83±0.38	0.77±0.27	0.040†

† p=0.040 for FIB-4 does not survive Bonferroni correction (adjusted threshold ≈0.006). Triglycerides and FIB-4: Mann-Whitney U test; others: independent samples t-test. TyG: triglyceride–glucose; FIB-4: fibrosis-4; AST: aspartate aminotransferase; ALT: alanine aminotransferase.

### Multivariable analysis

The TyG index was standardized prior to analysis (mean=4.826, SD=0.227; the observed group difference of 0.226 units corresponds to 1.00 SD). In multivariable logistic regression, TyG was the only independent predictor of nephrolithiasis (adjusted OR per 1 SD=3.52; 95%CI 2.48–5.00; p<0.001). Male sex was also independently associated (OR 11.42; 95%CI 5.67–23.01; p<0.001). Age (OR 0.999; p=0.963) and FIB-4 (OR 0.885; p=0.809) were not independently associated. The model achieved 83.3% overall accuracy (Nagelkerke pseudo-R^2^=0.511; Table 3).

**Table 3:** Multivariable logistic regression for nephrolithiasis.

Variable	Adjusted OR	95%CI	p-value
TyG index (per 1 SD)	3.52	2.48–5.00	<0.001
Sex (male vs. female)	11.42	5.67–23.01	<0.001
Age (per year)	0.999	0.967–1.033	0.963
FIB-4 index	0.885	0.328–2.383	0.809

Enter method; TyG standardized (per 1 SD=0.23 units). Overall accuracy: 83.3%; Nagelkerke pseudo-R^2^=0.511. OR: odds ratio; CI: confidence interval; TyG: triglyceride–glucose; SD: standard deviation; FIB-4: fibrosis-4.

### Receiver operating characteristic analysis

ROC analysis (Figure 1) demonstrated moderate discriminatory ability of the TyG index (AUC=0.779; 95%CI 0.729–0.827; p<0.001). The FIB-4 index showed no meaningful discriminatory ability (AUC=0.545; 95%CI 0.479–0.606; p=0.167). A TyG cutoff of 4.69 yielded 79.5% sensitivity and 61.2% specificity, with a positive predictive value of 89.8% and a negative predictive value of 40.9%. The high positive predictive value reflects the high prevalence of nephrolithiasis in this clinical sample (∼85.5%) and may not generalize to lower-prevalence populations, whereas the low negative predictive value indicates limited rule-out performance.

**Figure 1:**
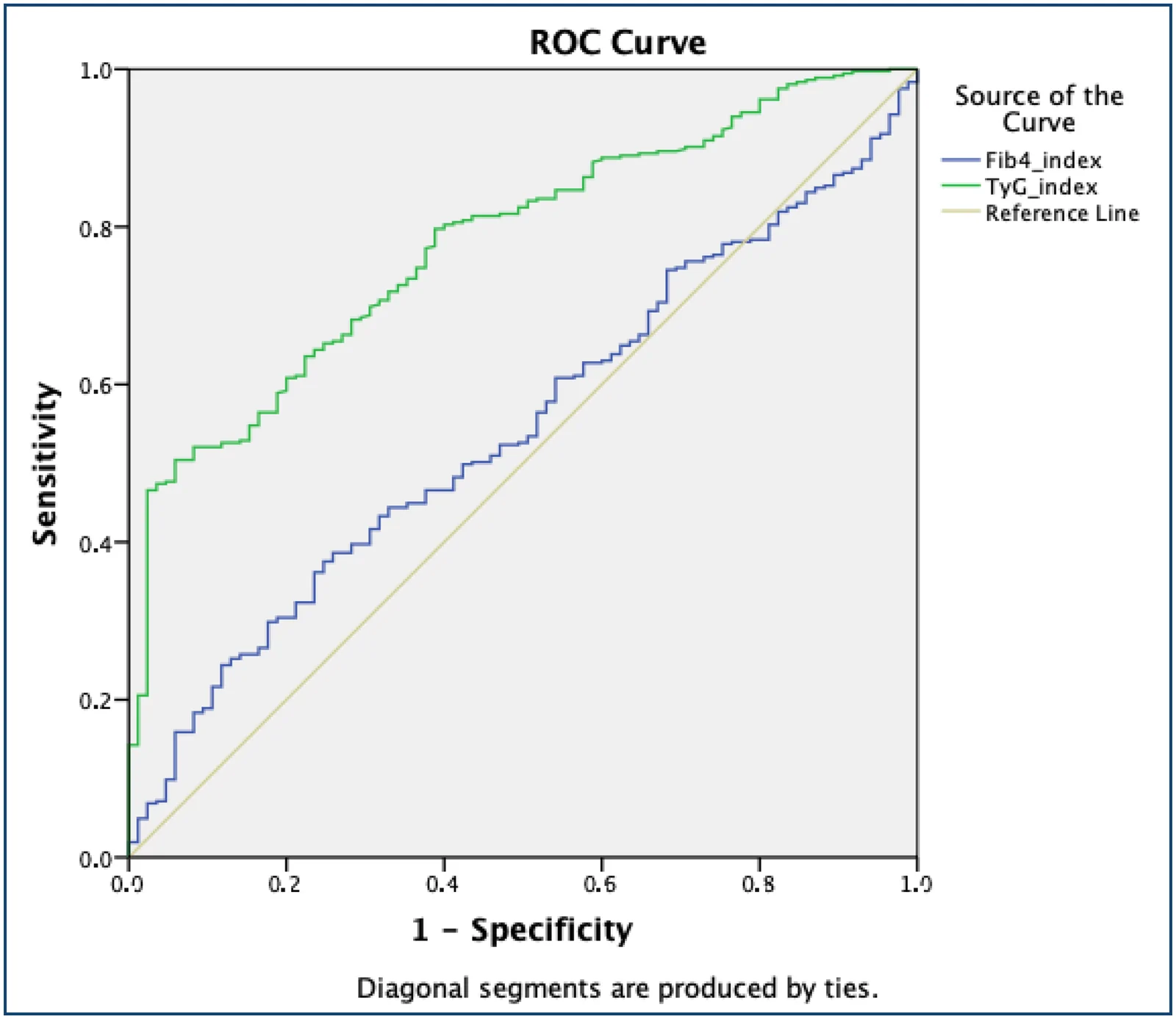
Receiver operating characteristic curve of the triglyceride–glucose and fibrosis-4 index for predicting nephrolithiasis. The area under the curve was 0.779 for triglyceride–glucose (p<0.001), while the area under the curve was 0.545 for fibrosis-4 (p=0.375).

### Sex-stratified analysis

Sex-stratified analysis demonstrated that the association between TyG and nephrolithiasis remained significant in both males (OR 5.14; 95%CI 2.27–11.66; p<0.001; AUC=0.880) and females (OR 3.09; 95%CI 2.09–4.56; p<0.001; AUC=0.761). The sex×TyG interaction term was not statistically significant (OR 1.95; 95%CI 0.81–4.68; p=0.135), indicating no significant effect modification by sex.

## DISCUSSION

This cross-sectional study demonstrates that both TyG and FIB-4 indices are elevated in individuals with nephrolithiasis, supporting the concept of kidney stone disease as a manifestation of systemic metabolic dysfunction. The TyG index showed a stronger cross-sectional association and superior discriminative performance.

The association between elevated TyG and nephrolithiasis is consistent with previous large population-based studies^10,12,13^. Each 1 SD increase in TyG (0.23 units, approximately the observed group difference) was independently associated with a 3.5-fold increase in the odds of nephrolithiasis. This standardized estimate is more clinically interpretable than raw-unit odds ratios and is consistent with the graded risk relationships reported in NHANES-based and Chinese population-based case–control studies^10,12^. Our TyG cutoff of 4.69 aligns with the range reported in prior studies (approximately 4.5–5.0).

The TyG–nephrolithiasis association was consistent across both sexes (male OR 5.14, AUC=0.880; female OR 3.09, AUC=0.761) with no significant sex interaction (p=0.135), in keeping with NHANES-based findings^10^. The somewhat higher OR in males may reflect the small male control group (n=14) and should be interpreted cautiously. The marked male predominance among stone formers (69.6 vs. 37.6%) is consistent with established epidemiology^2^.

Mechanistically, insulin resistance provides a plausible biological link. Impaired renal ammoniagenesis and reduced urinary citrate excretion lead to lower urinary pH, promoting calcium oxalate and uric acid crystallization^14,15^. Insulin resistance is also associated with tubular inflammation, oxidative stress, and altered electrolyte handling, which may further facilitate lithogenesis^16^.

The modest elevation of FIB-4 among stone formers aligns with emerging evidence linking MASLD and nephrolithiasis^17,18^. Meta-analyses have confirmed a significant association between MASLD and kidney stone disease, while imaging-based investigations have demonstrated increased hepatic steatosis prevalence among nephrolithiasis patients, supporting shared metabolic pathways^19^. However, the FIB-4 difference (0.83 vs. 0.77) is clinically negligible—both values are within the low-risk range (<1.45)—the difference did not survive Bonferroni correction, and FIB-4 was not independently associated (OR 0.885; p=0.809). Its AUC of 0.545 indicates no meaningful discriminatory ability beyond chance, suggesting that hepatic fibrotic burden is not an independent driver of nephrolithiasis in this population.

Notably, aminotransferases did not differ significantly between groups, highlighting the value of composite indices such as FIB-4 in detecting subclinical metabolic alterations, even when individual components are unremarkable. This study provides real-world clinical data regarding the association of insulin resistance-based and fibrosis-based indices with nephrolithiasis.

Several limitations should be acknowledged. The cross-sectional design precludes causal inference. The unequal case-to-control ratio (∼6:1) reflects the clinical setting of a tertiary referral urology center and may inflate sex-specific OR estimates and affect PPV/NPV generalizability; residual selection bias cannot be excluded. Dietary factors, urinary biochemistry, stone composition, and stone location were not systematically available. BMI was available only in the stone group (n=209; mean 28.69±4.21 kg/m^2^) and absent in controls. Although hepatic steatosis was recorded descriptively from available imaging reports, these data were incomplete across the study population and therefore were not incorporated into multivariable analyses. Medication use and family history of stones were not recorded. Advanced imaging for liver fibrosis validation also was not performed.

Future prospective studies should evaluate whether TyG predicts incident stone formation and recurrence, and whether insulin resistance-targeted interventions reduce stone risk.

## CONCLUSION

In this cross-sectional case–control study, each 1 SD increase in TyG index was independently associated with a 3.5-fold increase in the odds of nephrolithiasis (adjusted OR 3.52; 95%CI 2.48–5.00; p<0.001), with moderate discriminatory ability (AUC=0.779). This association was consistent across both sexes with no significant sex interaction. FIB-4 did not demonstrate independent association or meaningful discriminatory ability. Prospective longitudinal studies are needed to determine whether TyG predicts incident nephrolithiasis and to clarify its potential clinical applicability.

## Data Availability

The datasets generated and/or analyzed during the current study are available from the corresponding author upon reasonable request.
